# Adolescent female rats recovered from the activity‐based anorexia display blunted hedonic responding

**DOI:** 10.1002/eat.23752

**Published:** 2022-06-11

**Authors:** Matthew M. Hurley, Ashraf N. Nawari, Victoria X. Chen, Shannon C. O'Brien, Aliasgher I. Sabir, Ethan J. Goodman, Lucas J. Wiles, Aditi Biswas, Sean Andrew Aston, Seva G. Khambadkone, Kellie L. Tamashiro, Timothy H. Moran

**Affiliations:** ^1^ Department of Psychiatry & Behavioral Sciences The Johns Hopkins University School of Medicine Baltimore Maryland USA

**Keywords:** animal models, anorexia nervosa, eating disorders, hedonics, machine learning

## Abstract

**Objective:**

As patients with anorexia nervosa tend to “like” palatable tastants less than controls, we set out to model this preclinically by using the taste reactivity test (TRT) to assess hedonic state in rats following weight restoration from a bout of activity‐based anorexia (ABA).

**Method:**

Female rats (*n* = 31) were surgically implanted with an intraoral catheter, which allowed experimenters to assess baseline TRT to six tastants. Following baseline TRT, animals were either exposed to the activity‐based anorexia condition (ABA; 1.5HR chow/ad lib wheel until 25% weight loss), kept sedentary (SED; ad lib chow/locked wheel), given access to running wheels with ad lib chow access (RW; ad lib chow/wheel), or were body weight matched to the ABA group (BWM; restricted chow/locked wheel). Following 25% weight loss, wheels were locked and food returned to ABA rats. Paired RW groups had their wheels locked and paired BWM rats were given ad lib access to food. Animals were given 10 days to recover prior to a second TRT. Videos were analyzed for liking (tongue protrusions) and disliking (gape) behaviors.

**Results:**

The ABA group displayed a significant within‐subject reduction in cumulative lick responses to water and 1 M sucrose. Additionally, we found the SED and ABA group displayed a significant within‐subject reduction in cumulative lick responses to .1 M sucrose. Positive hedonic responses did not decline in either the BWM or the RW groups.

**Discussion:**

The data show a novel phenomenon that a history of ABA results in an anhedonia phenotype that mirrors aspects of AN.

**Significance statement:**

Patients recovered from anorexia nervosa report anhedonia, or the lack of pleasure in consuming palatable foods. Unfortunately, the biological mechanism underpinning anhedonia in anorexia nervosa is not well understood. The current study assessed hedonic state in adolescent female rats prior to and 10 days recovered following the activity‐based anorexia paradigm. Age‐matched, running wheel‐matched and body weight‐matched control groups were also tested at the same time points.

## 1 INTRODUCTION

Patients diagnosed with anorexia nervosa (AN) have been documented as having one of the highest mortality rates among psychiatric disorders (Arcelus et al., 2011). AN is a devastating eating disorder characterized by excessive exercise, self‐starvation and significant weight loss (Beumont et al., 1978; Castellini et al., 2013; Gaudio et al., 2014). Unfortunately, there are no effective therapeutic approaches outside of cognitive behavioral therapy and refeeding to combat the severity as well as the threat of relapse to AN. In an effort to identify potential biological mechanisms that may explain the consequences of engaging in the behaviors underlying AN, it is important to model the symptoms. Anhedonia, or the lack of pleasure in experiencing rewarding stimuli, is a prominent symptom observed in acutely ill, acutely recovered and long‐term recovered AN patients (Boehm et al., 2018). Others have hypothesized this long‐lasting impairment in hedonic responding may be a driving force behind the high rate of relapse observed in these patients (Zipfel et al., 2000). Unfortunately, the biological mechanisms underlying anhedonia in this patient population are largely unknown.

First described in 1967, activity‐based anorexia (ABA) is a preclinical animal model that combines time‐limited feeding (1.5‐2HR access to food/day at the onset of dark cycle) with ad lib wheel access. This combination drives hyperactivity, voluntary food restriction and results in rapid weight loss (Routtenberg & Kuznesof, 1967). Over the years, this model has been shown to reproduce several consequences of AN, such as impairments in cognitive function (Boersma et al., 2016; Lamanna et al., 2019). Here, we used this model to study the impact of ABA experience on hedonic responses in adolescent female rats. To asses hedonics, we used the well‐established taste reactivity test (TRT), which objectively measures orofacial responses that represent “liking”/“disliking” responses that are evolutionarily conserved with similar responses displayed by human infants, monkeys, and rodents (Grill & Norgren, 1978). In brief, this rodent technique involves surgically implanting an intraoral catheter which allows the researcher to infuse various tastants directly into the oral cavity. Animals are filmed through a transparent floor and these videos are scored frame‐by‐frame for evolutionarily conserved orofacial responding such as tongue protrusions which is considered a “liking” response or a “gape” response which is considered a display of “disliking” or aversion. The different responses are carefully characterized in Grill & Norgren's original study and this method has since been extensively used to study hedonic responding (Berridge, 2000; Ho & Berridge, 2014; Roitman et al., 2005). In the present study, we assessed orofacial responding to six tastants at baseline and after 10 days of weight recovery in rats exposed to the ABA paradigm. We compared the data with those from control groups that were either sedentary, body weight matched to the ABA rats or had access to a running wheel with ad lib access to chow.

## 2 MATERIALS AND METHODS

### 2.1 Animals

**FIGURE 1:**
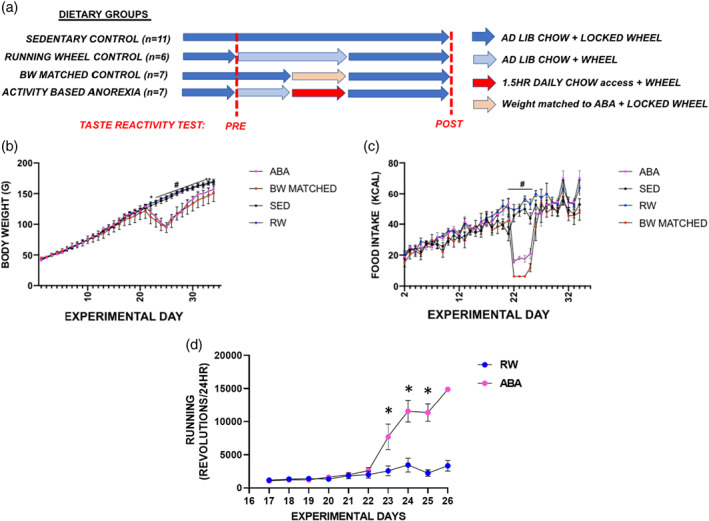
Adolescent female rats were offered time‐limited feeding (1.5 HR/day) with ad lib access to a running wheel produces behaviors that mirror symptoms of anorexia nervosa. (a) Experimental design of novel taste reactivity test + activity‐based anorexia paradigm. (b) 24‐hour food intake measurements throughout the duration of the study. ABA animals have significantly lower food intake compared with the SED or RW group while the animals are actively on the paradigm. (c) Daily body weight measurements in sedentary controls (SED), running wheel controls (RW) and activity‐based anorexia (ABA) throughout the duration of the experiment. ABA animals lost a significant amount of body weight compared with SED and RW groups during the ABA paradigm. (d) Cumulative 24‐hour wheel revolutions in the ABA and RW group over the duration of the study. Wheel running was significantly increased in the ABA animals during time‐limited feeding + ad lib wheel. Data are presented ± SEM, *p < .05 post hoc (Tukey) ABA versus SED or RW; #*p* < .05 post hoc (Tukey) ABA versus SED. ABA, activity‐based anorexia; RW, wheel habituation; S, surgery; TR, taste reactivity

Female Sprague–Dawley rats were 22 days old upon arrival (*n* = 31; Envigo; Frederick, MD) and were divided into four experimental groups (Figure 1a): sedentary control (SED; *n* = 11; ad lib + locked wheel for the duration of the study), running wheel control (RW; *n* = 6; ad lib chow + locked wheel from experimental day 1–15; ad lib chow and wheel access from experimental day 16–26; ad lib chow + locked wheel from experimental day 26–35), body weight‐matched control (BWM; *n* = 7; ad lib chow + locked wheel from experimental day 1–21; restricted from experimental day 22–26 + locked wheel; ad lib chow + locked wheel from experimental day 26–35) and activity‐based anorexia (ABA; *n* = 7; ad lib chow and a locked wheel from experimental day 1–15; ad lib chow and wheel from experimental day 16–21; 1.5HR chow and ad lib wheel from experimental day 22–26; ad lib chow and locked wheel from experimental day 26–35). One sedentary animal is included in the food intake and body weight data, but not in the TRT data as there were complications with the intraoral catheter. The data included in the following manuscript was conducted in two cohorts. Cohort one consisted of the groups SED (*n* = 5), RW (*n* = 6), and ABA (*n* = 7). Once we knew the body weights of the ABA group, we conducted a second cohort with SED (*n* = 6) and a group body weight matched (*n* = 7) to the ABA group. All rats were singly housed in conventional tub cages equipped with a running wheel under a standard 12 h:12 h light:dark cycle. Animals had ad lib access to a nutritionally balanced standard chow (Envigo Teklad Global 18% Protein Rodent Diet; 3.1 kcal/gram) and water unless otherwise indicated. Food intake and body weight were measured once daily prior to the onset of the dark cycle. All procedures were approved by the Johns Hopkins University Animal Care and Use Committee.

### 2.2 Surgery

Animals were allowed to acclimate to the colony room for 48 hrs. Animals were then anesthetized with isoflurane and implanted with an intraoral catheter placed to the left of the upper first molar. Intra oral catheters were made with 3.5 inches of polyethylene tubing at I.D. .86 mm/O.D. 1.27 mm (BD Intramedic, Sparks, MD). The catheter was then carefully navigated under the skin and emerged behind the animal's ear. Immediately following surgery, animals received a single intramuscular injection of Banamine (1.1 mg/kg) as an analgesic. Animals were then given 5 days to recover with ad lib access to chow and water. Intraoral catheters were flushed once daily with water.

### 2.3 Taste reactivity test

Following recovery from surgery, all animals were habituated to the TRT chamber (PlexiGlass cylinder 30 cm in diameter × 30 cm in height) for 15 min/day over 3 days. The floor of the chamber was made of clear PlexiGlass allowing for the animal's behavioral responses to be recorded by a video camera positioned underneath the testing chamber. On test day, the animal was placed in the chamber for six 1‐min trials (1 ml delivered per tastant) of water, .01 M sucrose, .1 M sucrose, 1.0 M sucrose, .0003 M quinine and finally .003 M quinine, presented in that order, with less than 40–60 s in between trials. Between each trial, the animals' mouth was washed with water and the chamber was cleaned with a 70% ethanol solution. Previous work demonstrates prior sucrose exposure can impact subsequent taste reactivity responding to quinine and vice versa (Suárez et al., 2017). Therefore, we recognize a limitation to our design here was to not randomize the order in which the tastants were delivered and future studies will take this into account. This TRT was conducted at two timepoints: baseline (“Pre” test) when the animals were 35–36 days old (experimental day 14 or 15) and following the ABA and recovery period (“Post” test) at 56–58 (experimental day 33, 34, or 35) days old (Figure 1a).

### 2.4 Machine learning‐assisted taste reactivity scoring

**FIGURE 2:**
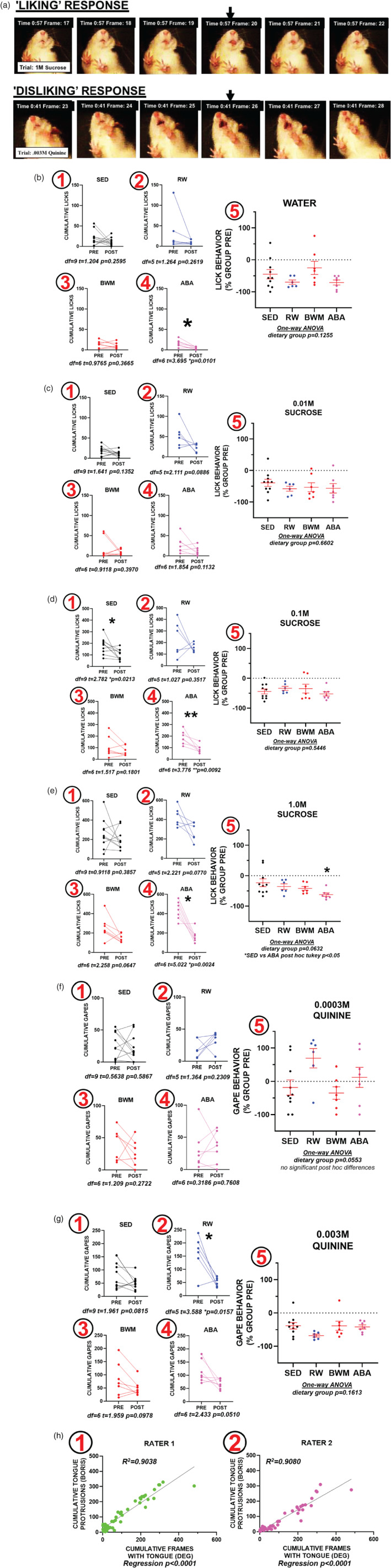
Taste reactivity data. (a) Representative images of frame‐by‐frame orofacial “liking” (top) and “disliking” (bottom) responses. (b) When comparing cumulative lick responses to water via paired t‐test for each group (b1‐4), we found only the ABA group showed a significant reduction in responding (b4). This was not observed in the SED (b1), RW (b2), or BWM (b3) groups. When comparing the percent change in lick behavior from group baseline (b5), we found no significant differences between the groups when analyzed by one‐way ANOVA. (c) When comparing cumulative lick responses to .01 M sucrose via paired *t‐test* (c1‐4), we found none of the groups demonstrated a significant change. When comparing the percent change in lick behavior from group baseline (c5) we found no significant differences between the groups when analyzed by one‐way ANOVA. (d) Paired t‐test results comparing pre and post cumulative lick responses to .1 M sucrose show the SED (d1) and ABA (d4) groups show a significant reduction in responding. This within‐subject difference was not observed in the RW (d2) or the BWM (d3) groups. When comparing the percent change in lick behavior from group baseline (d5) we found no significant differences between the groups when analyzed by one‐way ANOVA. (e) Paired t‐test results comparing pre and post cumulative lick responses to 1.0 M sucrose show the ABA (e4) animals are the only group with a significant reduction. This within‐subject reduction in lick responding was not observed in the SED (e1), RW (e2), or BWM (e3) groups. When comparing the percent change in lick behavior from group baseline (e5), we found a significant post hoc difference such that the ABA group showed a larger negative change in responding compared with the SED group. (f) Paired t‐test comparing cumulative gape behavior to .0003 M quinine at the pre and post timepoint in all four groups. There were no within‐subject differences in the SED (f1), RW (f2), BWM (f3), or ABA (f4) groups. When comparing the percent change in lick behavior from group baseline (f5) we found no significant differences between the groups when analyzed by one‐way ANOVA. (g) Paired t‐test comparing cumulative gape behavior to .003 M quinine at the pre and post timepoints in all four groups. We found a significant within‐subject reduction in gape behavior in the RW group (g2), but not in the SED (g1), BWM (g3), or ABA (g4) groups. When comparing the percent change in lick behavior from group baseline (g5), we found no significant differences between the groups when analyzed by one‐way ANOVA. (h) Regression analysis comparing DEG finalized labels versus labels provided by two raters that scored videos using a more conventional method. We found regression with an r‐squared value of .9038 for rater 1 (h1) and .9080 for rater 2 (h2). Data is presented as individual animals or mean ± SEM. *p < .05 paired t‐test or one‐way ANOVA post hoc Tukey comparison

Videos recorded during the TRT (*n* = 360) were analyzed using a machine learning pipeline known as DeepEthogram (DEG; Bohnslav et al., 2021). We have previously published the use of this software to analyze orofacial responding during the TRT test (Hurley et al., 2021). This software uses convolutional neural networks (CNN) to perform behavioral classification from raw pixels and user input. After installation (http://github.com/jbohnslav/deepethogram), experimenters loaded a set of test videos (*n* = 5) and started training the *flow generator* CNN, which requires no user input. The architecture of the flow generator CNN is *MotionNet*, which contains 45.8 million trainable parameters (Simonyan & Zisserman, 2014; Zhu et al., 2019). This model takes the video and distills the motion in between each frame into a data file known as an optical flow. Appetitive (liking) and aversive (disliking) responses were categorized using the techniques outlined by Chan et al. (2016) and Grill and Norgren (1978). While the flow generator trains, the experimenter labeled frames either background (0), lick (1), paw lick (2), gape (3), paw flail (4), and wet dog shake (5). Next the output of the flow generator and the user provided labels are used to train the feature extractor. The architecture of the feature extractor is ResNet50 (He et al., 2016), which uses a hidden two‐stream CNN that extracts a low‐dimensional set of spatial (RGB frames) and temporal (optic flow frames) features to model the probability of a particular behavior being present. Once the feature extractor training was complete, it was used to predict on new videos, which were error corrected and used to re‐train the model. Once all 360 videos were labeled, they were randomly divided into training and validating datasets and used to train DEG, one time from the pretrained weights. The human‐corrected labels, DEG predictions and model parameters are available at the following link: https://www.dropbox.com/sh/3ar5tt0vgc4he26/AABz0ZxoSA7s4wnQqzqeE5lfa?dl=0. As DEG is an open‐source software, readers can use our large repository of labeled videos to test DEG model performance with different parameters than those used in this manuscript. Additionally, readers can use our pretrained weights as a starting point to add in new videos to improve the generalizability of this TRT model. Finally, we wanted to validate the use of DEG in scoring TRT by comparing DEG labels to scoring collected using more conventional methods. A subset of the videos (*n* = 52) was scored by two raters using the Behavioral Observation Research Interactive Software (BORIS; Friard & Gamba, 2016). The raters were blinded to experimental conditions and watched the videos for tongue protrusions at 1/10th speed in one take (no pausing). These values were then plotted against the DEG finalized labels (Figure 2h).

### 2.5 Activity‐based anorexia, running, body weight groups

Following baseline TRT, animals in the ABA group (*n* = 7) and the RW control group (*n* = 6) were given ad lib access to their running wheels and food for 5 days starting on experimental day 16 to habituate to the running wheel. Following habituation, animals in the ABA group were placed on a time‐restricted feeding schedule (1.5HR food access at onset of the dark cycle, 24HR ad lib water access) with ad lib wheel access until the animals lost 25% of their original body weight. This was done 90 min into the dark cycle on experimental day 21. Of the seven animals in the ABA group, one animal met the weight loss criterion by 3 days of ABA, five animals by 4 days, and one animal by 5 days. As each ABA animal reached their 25% body weight loss criterion, their running wheel was locked and they were provided with ad lib access to food. The BWM group was given 2 g of chow per day at the onset of the dark cycle which resulted in weight loss similar to that exhibited by the ABA group. Paired participants from the BWM control group were also placed in recovery with a locked wheel and ad lib food access after 3, 4, or 5 days of experimental conditions corresponding to their paired ABA subject. The RW group was all stopped when the last ABA animal reached criterion. All animals then went through a 10‐day recovery period and were then tested in the TRT a second time (“Post” test). We and others have previously demonstrated that most of the animals exposed to the ABA paradigm are susceptible while a smaller subset are resistant to achieving the 25% weight loss (Milton et al., 2018, Hurley et al., 2022). In the current cohort, we did not have any resistant animals, which is atypical. We attribute this to the experiment being conducted on a limited number of outbred genetically diverse animals. Future studies replicating this work should consider using a larger initial cohort of ABA animals to assess if the phenomena described in this manuscript are specific to animals exhibiting the prone phenotype or generalize to resistant animals as well.

### 2.6 Statistics and availability of data

Food intake, body weight, and wheel running data are presented as mean ± standard error of the mean and analyzed by two‐way repeated measure ANOVA. Tukey analysis was used for *post hoc* group comparisons and *p* < .05 was considered statistically significant. Taste reactivity data were analyzed by two‐tailed paired students *t*‐test. Additionally, to compare the magnitude of change among the groups, we plotted each animal's post data as a percent of the group average baseline and then compared these via one‐way ANOVA, for each tastant. Every one‐way ANOVA included post hoc Tukey comparisons and were reported if *p* < .05. All statistics and graphs were plotted on PRISM (GraphPad Software, San Diego, CA). The DEG model (including 360 videos, labels, predictions and weights) can be found at the Dropbox link provided in the manuscript. Any other data that support the findings of this study are available on request from the corresponding author.

## 3 RESULTS

A two‐way repeated measure ANOVA of daily body weights revealed a significant interaction of experimental group over time (Figure 1b; f[99,924] = 12.97, *p* < .0001). The ABA paradigm was started on experimental day 21. Post hoc Tukey t‐tests revealed that the body weights of the ABA or BWM group were significantly lower than those of the SED or RW group 24 h into the ABA period on experimental day 22 (Figure 1b; *p* < .05 post hoc Tukey t‐test). When analyzing daily food intake by two‐way repeated measure ANOVA, we also found an interaction of experimental group over time (Figure 1c; f[96, 864] = 7.665, *p* < .0001). Post hoc Tukey t‐tests revealed the food intake of the ABA or BWM group was significantly lower than intake in the RW or SED animals during experimental day 22–25 (Figure 1c; *p* < .05 post hoc Tukey *t*‐test). Finally, when examining 24‐h cumulative wheel revolutions (Figure 1d), we found a significant effect of experimental group and time (GROUP f(1,122) = 19.727, *p* < .001; TIME f(9,122) = 18.123, *p* < .001). There were no differences in wheel revolutions between RW and ABA at any timepoint during baseline (experimental day 17–21). However, once food access was limited to 1.5 HR per day, the ABA group greatly increased their number of wheel revolutions relative to the RW group's activity (Figure 1d; post hoc Tukey *t*‐test; ABA vs. RW; *p* < .001).

Involuntary infusion of tastants into the adolescent female rat oral cavity results in evolutionary conserved and objective orofacial responses that represent “liking” and “disliking” responses (Figure 2a). Here, we measured orofacial responding to six tastants at baseline and following 10 days recovery from the experimental conditions. One milliliter of each tastant (water, .01 M sucrose, .1 M sucrose, 1.0 M sucrose, .0003 M quinine, .003 M quinine) was delivered over a 60 s period and the data were analyzed frame‐by‐frame for orofacial responses. We restricted our analysis of orofacial responding to lick behavior, representing “liking” and gape behavior, representing “disliking.”

**FIGURE 3:**
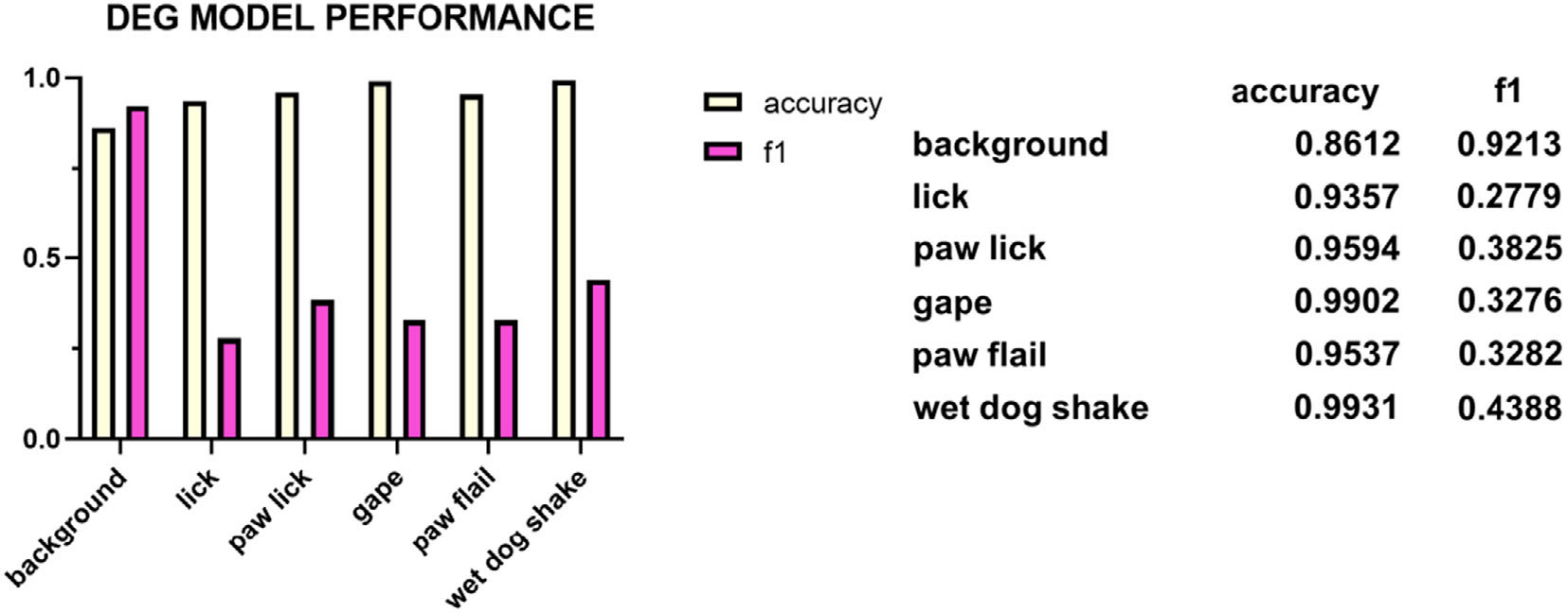
Machine learning model performance identifying orofacial responding. In total, 360 videos labeled for orofacial behaviors were randomly divided into training and validating dataset. (Left) Accuracy and F1 score on validating dataset after 19 epochs (cycles) of training identifying background, lick, paw lick, gape, paw flail and wet dog shake. (Right) Table showing exact values of accuracy and F1 graphed in the left panel

When examining cumulative “lick” responses elicited to water, we found that only the ABA group displayed a significant within‐subject decrease in cumulative lick responses (Figure 2b‐4; *paired t*‐test; *t* = 3.695; df = 6; *p* = .0101). This within‐subject effect was not observed in the SED group (Figure 2b‐1; *paired t*‐test; *t* = 1.204; df = 9; *p* = .2595), RW group (Figure 2b‐2; *paired t*‐test; *t* = 1.264; df = 5; *p* = .2619) or BWM group (Figure 2b‐3; *paired t*‐test; *t* = .9765; df = 6; *p* = .3665). We found no group differences when comparing the PRE to POST percent change in lick behavior (Figure 2b‐5; *one‐way ANOVA*, *p* = .1255).

For .01 M sucrose, we found no significant within‐subject differences in the SED group (Figure 2c‐1; *paired t*‐test; *t* = 1.641; df = 9; *p* = .1352), the RW group (Figure 2c‐2; *paired t*‐test; *t* = 2.111; df = 5; *p* = .0886), the BWM group (Figure 2c‐3; *paired t*‐test; *t* = .9118; df = 6; *p* = .3970) or the ABA group (Figure 2c‐4; *t* = 1.854; df = 6; *p* = .1132). Additionally, we found no group differences when comparing the PRE to POST percent change in lick behavior (Figure 2c‐5; *one‐way ANOVA*, *p* = .6602).

For .1 M sucrose, we found the SED group (Figure 2d‐1; *paired t*‐test; *t* = 2.782; df = 9; *p* = .0213) and the ABA group (Figure 2d‐4; *paired t*‐test; *t* = 3.776; df = 6; *p* = .0092) displayed a significant within‐subject reduction in responding. This reduction was not observed in the RW (Figure 2d‐2; *paired t*‐test; *t* = 1.027; df = 5; *p* = .3517), or BWM groups (Figure 2d‐3; *paired t*‐test; *t* = 1.517; df = 6; *p* = .1801). We found no group differences when comparing the PRE to POST change in lick behavior (Figure 2d‐5; *one‐way ANOVA*, *p* = .5446).

At the highest concentration of sucrose tested (1 M), we found that only the ABA group had a significant within‐subject reduction in cumulative “lick” responses (Figure 2e‐4; *paired t*‐test; t = 5.022; df = 6; *p* = .0024). This within‐subject reduction in appetitive responding to 1 M sucrose was not observed in the SED group (Figure 2e‐1; *paired t*‐test; *t* = .9118; df = 9; *p* = .3857), the RW group (Figure 2e‐2; *paired t*‐test; *t* = 2.221; df = 5; *p* = .0770), or the BWM group (Figure 2e‐3; *paired t*‐test; *t* = 2.258; df = 6; *p* = .0647). When comparing the PRE to POST percent change in lick behavior, although we did not find a main effect between the groups (Figure 2e‐5; *one‐way ANOVA*; *p* = .0632); post hoc testing revealed that the ABA group had a significantly greater reduction in lick behavior compared with the reduction displayed by the SED group (Figure 2e‐5; post hoc *Tukey*; *p* < .05).

To test animals orofacial responding to a bitter taste, we conducted a .0003 M quinine and .003 M quinine trial. Therefore, instead of analyzing cumulative lick frames, we analyzed cumulative gape frames. When examining within‐subject changes in cumulative gape responses we found no significant differences in the SED (Figure 2f‐1; *paired t*‐test; *t* = .5638; df = 9; *p* = .5867), RW (Figure 2f‐2; *paired t*‐test; *t* = 1.364; df = 5; *p* = .2309), BWM (Figure 2f‐3; *paired t*‐test; *t* = 1.209; df = 6; *p* = .2772) or ABA group (Figure 2f‐4; *paired t*‐test; *t* = .3186; df = 6; *p* = .7608). We found no differences between the groups when comparing the percent change in gape behavior from the PRE timepoint (Figure 2f‐5; *one‐way ANOVA*, *p* = .0553). There were no post hoc differences in this one‐way ANOVA. We found the RW group displayed a significant within‐subject reduction in cumulative “gape” frames to .003 M quinine (Figure 2g‐2; *paired t*‐test; *t* = 3.588; df = 5; *p* = .0157). This was not observed in the SED (Figure 2g‐1; *paired t*‐test; *t* = 1.961; df = 9; *p* = .0815), BWM (Figure 2g‐3; *paired t*‐test; *t* = 1.959; df = 6; *p* = .0978) or ABA groups (Figure 2g‐4; *paired t*‐test; *t* = 2.433; df = 6; *p* = .0510). Finally, we found no differences between the groups when comparing the PRE to POST percent change in gape behavior (Figure 2g‐5; *one‐way ANOVA*, *p* = .1613).

To validate the use of DEG as an approach to scoring TRT, two raters, blinded to group status and tastant, analyzed a subset of these videos (*n* = 52) using a more conventional method (Figure 2h). We found the DEG labels highly correlated to both raters' labels (Figure 2h‐1, *r* ^2^ = .9038; Figure 2h‐2, *r* ^2^ = .9080; *regression analysis*; *p* < .0001).

Once all 360 videos were labeled, these were used to train DEG one time from the pretrained weights. For the purposes of this manuscript, we report model performance by examining the accuracy score and F1 score in the validating dataset. Accuracy is calculated by dividing the total number of frames identified as a true‐positive and true‐negative for a given behavioral class by the sum of the total frames. F1 score is a weighted average between 0 and 1 that takes into consideration the rate of true‐positives and false‐negatives. A F1 score of 1 is perfect performance, while 0 is extremely poor performance. At the end of training, DEG detected “background” with .8612 accuracy and .9123 F1 score; “lick” with .9357 accuracy and .2779 F1 score; “paw lick; with .9594 accuracy and .3825 F1 score; “gape” with .9902 accuracy and .3276 F1 score; “paw flail” with .9537 accuracy and .3282 F1 score; and finally “wet dog shake” with .9931 accuracy and .433 F1 score (Figure 3). Although DEG demonstrated high accuracy in detecting our behaviors of interest when they are present, there is over predicting false‐positives and false‐negatives, which leads to a diminished F1 score.

## 4 DISCUSSION

Long‐lasting anhedonia is observed in acutely ill and long‐term recovered AN patients and may be a driving force for relapse in these patients. We and others have used the preclinical ABA paradigm to model aspects of AN. We previously demonstrated that animals with a history of ABA do not differ from sedentary controls in their consumption of sucrose in a brief access taste test, but do differ in measures of cognitive function (Boersma et al., 2016) and are more sensitive to a conditioned taste aversion paradigm than controls (Liang et al., 2011). More recently, we demonstrated animals prone to the ABA paradigm displayed fewer lick responses to a 1 M sucrose TRT conducted at maximum weight loss compared with animals resistant to the paradigm (i.e., did not lose 25% body weight). We did not find retest differences between prone and resistant animal orofacial responding at the 10‐day recovered timepoint (Hurley et al., 2022). This led to the current study comparing how a history of ABA impacts orofacial responding compared with three control groups. We measured orofacial responses to palatable and aversive tastants in adolescent female rats prior to and after 10 days recovery from experience with ABA, running alone, food restriction alone, or sedentary conditions.

We demonstrate that 1.5HR chow access with ad lib running wheel access results in ABA. Animals in the ABA paradigm exhibited limited food intake, excessive wheel running, and rapid weight loss (Figure 1). When examining cumulative lick responses to a high concentration of sucrose (1 M) as well as water, we found that only animals with a history of ABA showed lower “liking” responses during the post ABA TRT (Figure 2b,e), a phenomena not observed in SED, BWM, or RW control groups. This finding suggests the reduction in sucrose responding could be an additive effect of the activity in the running wheel and the reduced food intake. An alternative conclusion may be that there is a motor‐based deficit, rather than specifically anhedonia, as the ABA group also shows a reduction to water which is a neutral tastant (Figure 2b). However, it is unlikely that it is a motor deficit as this phenomena would have been observed in all the tastants. When comparing the percent change from baseline, we found that at 1 M sucrose, the ABA group had a significantly larger reduction compared with that of the SED group (Figure 2e). Although this finding suggests that the ABA group is showing signs of anhedonia, it is important to note that the ABA group had a greater PRE response than the other groups and therefore had more room to decrease. When examining cumulative lick responses at .1 M sucrose, we found that the ABA and SED groups displayed significant reductions in responding (Figure 2d). The reduction in SED lick responses is consistent with data from others that found that positive orofacial responding decreases with age in rats (Wilmouth & Spear, 2009). At .1 M sucrose, this same conclusion can be extended to the ABA group and therefore, this finding in ABA group is not evidence of anhedonia. However, at a higher concentration of sucrose (1.0 M), the magnitude of change is significantly greater than that in the SED group, supporting the interpretation of anhedonia. Thus, the anhedonia in AN may be modeled through combining the TRTs with the ABA paradigm. An unexpected finding was a significant reduction in gape responses to the high concentration of quinine observed only in the RW group (Figure 2g). As voluntary wheel running is rewarding for rodents (Heyse et al., 2015); it is possible these animals are now more tolerant of the aversive experience such as high concentration quinine.

A limitation to our study was the length of the ABA paradigm. All of the ABA animals in our experiment took a relatively short period of time to reach 25% weight loss. Others have used a modified ABA design to hold animals at maximum weight loss for longer periods of time (Frintrop et al., 2018). A future study should examine if prolonging animals' ABA period leads to a more dramatic shift in hedonic responding when 10 days recovered. Additionally, given that sucrose has caloric value, future studies could examine orofacial responding to saccharin, which is a palatable tastant without caloric value. Such a study would establish whether the phenomena is specific to the taste or depends upon the caloric load received.

Milton and colleagues (Milton et al., 2018) assessed hedonic drive using 2 bottle‐choice preference tests with either 1.5% sucrose or .02% saccharin over the time course of the ABA model. They found that only a small number of the ABA animals developed an anhedonic phenotype as measured by reduced preference for the sweetened solutions over the ABA paradigm. Furthermore, running wheel access alone was enough to decrease sweetened water preference. A key difference between the two studies is the method used to assess hedonics. The behavior of two‐bottle preference requires the animal to seek and consume the reward (mixed “wanting” and “liking” driven behavior), which is different than the *involuntary* delivery of tastant and quantifying response to the reward (i.e., “liking”) as was done in this study using the TRT. Another difference is that the two‐bottle preference tests were conducted during the ABA exposure. In addition, the criterion for ABA weight loss used by Milton and colleagues was 20% whereas our study used 25% weight loss as the criterion for removing the animal from the ABA model. Finally, we found a significant reduction in “liking” at a very high concentration of sucrose, which is different than the concentrations of the sweetened water used by Milton and colleagues. Taken together, these findings point to the complexity of the ABA paradigm. Multiple behavior tools are necessary to truly understand the ramifications of this paradigm on adolescent female rat homeostasis.

Anhedonia is a hallmark symptom of both major depressive disorder (MDD) and AN (Boehm et al., 2018; Lemke et al., 1999). Additionally, both of these disorders show significant impairments in oxidative state (Michel et al., 2012; Moyano et al., 1998; Zenger et al., 2004). MDD patients with the most severe impairment in oxidative state also displayed the most severe anhedonia (Michel et al., 2012). To our knowledge, there are no comparable studies yet examining these variables in a patient population diagnosed with AN. Recently, we demonstrated that adolescent female rats at maximum weight loss, but not 10‐days recovered, have deficits in plasma glutathione compared with sedentary controls (Hurley et al., 2021). This finding suggests that the ABA paradigm causes a transient state of heightened oxidative stress as glutathione is a primary antioxidant for the body. Given this finding and the data in the current manuscript, a future experiment could use microdialysis to repeatedly measure brain glutathione and taste reactivity at various timepoints of the ABA paradigm. A potential target for this microdialysis would be the medial prefrontal cortex as we previously found that animals prone to weight loss during ABA had lower astrocyte density in this region, even at the 10‐day recovered timepoint, compared with resistant animals (Hurley et al., 2022). This reduction in astrocytes is consistent with an earlier report that rats maintained on the ABA paradigm display cortical thinning and a reduction in astrocyte expression (Frintrop et al., 2018). Additionally, we have also reported increased mitochondrial fission in the medial prefrontal cortex of rats at maximum weight loss, but not when recovered from ABA (Hurley et al., 2021). Measuring cortex oxidative state and taste reactivity in the same animal would allow the assessment of whether the most severe ABA was also associated with anhedonia toward highly palatable tastants.

One possibility is that changes in the cortex may underlie the pathophysiology of AN as previous studies in patients with AN show significant thinning of cortex when acutely ill (Mainz et al., 2012; Seitz et al., 2016). Additionally long‐term recovered patients have hyperactive cortical responses to images of palatable foods (Frank et al., 2012). Taken together, these findings suggest that a transient increase in oxidative stress and cortical thinning when patients are acutely ill may underlie long term, persistent neurobiological changes, even in recovered patients. As AN patients suffer from a high rate of relapse, it is critical to continue using preclinical paradigms to better understand the biology underpinning this devastating disorder.

## AUTHOR CONTRIBUTIONS

**Matthew Hurley:** Conceptualization; data curation; formal analysis; funding acquisition; investigation; methodology; project administration; writing – original draft; writing – review and editing. **Ashraf N Nawari:** Data curation; investigation. **Victoria X Chen:** Investigation; methodology. **Shannon C. O'Brien:** Formal analysis; investigation; methodology. **Aliasgher I Sabir:** Investigation. **Ethan G. Goodman:** Investigation. **Lucas J Wiles:** Investigation. **Aditi Biswas:** Investigation. **Sean Andrew Aston:** Investigation. **Seva G. Khambadkone:** Methodology. **Kellie L. Tamashiro:** Conceptualization; project administration; resources; supervision; writing – original draft; writing – review and editing. **Timothy H. Moran:** Conceptualization; funding acquisition; project administration; resources; supervision; writing – original draft; writing – review and editing.

## CONFLICT OF INTEREST

The authors declare no conflict of interest in conducting this study.

## Data Availability

The DEG model (including 360 videos, labels, predictions and weights) can be found at the Dropbox link provided in the manuscript. Any other data that support the findings of this study are available on request from the corresponding author.
